# Mediating roles of leukoaraiosis and infarcts in the effects of unilateral carotid artery stenosis on cognition

**DOI:** 10.3389/fnagi.2022.972480

**Published:** 2022-09-29

**Authors:** Kuo-Lun Huang, Ting-Yu Chang, Yi-Ming Wu, Yeu-Jhy Chang, Hsiu-Chuan Wu, Chi-Hung Liu, Tsong-Hai Lee, Meng-Yang Ho

**Affiliations:** ^1^Department of Neurology, Linkou Chang Gung Memorial Hospital, Taoyuan City, Taiwan; ^2^College of Medicine, Chang Gung University, Taoyuan City, Taiwan; ^3^Department of Radiology, Linkou Chang Gung Memorial Hospital, Taoyuan City, Taiwan; ^4^Graduate Institute of Behavioral Sciences, Chang Gung University, Taoyuan City, Taiwan

**Keywords:** cognition, carotid artery stenosis (CAS), leukoaraiosis (LA), infarct, mediation analysis, white matter hyperintensities

## Abstract

**Background and objectives:**

Leukoaraiosis and infarcts are common in patients with carotid artery stenosis (CAS), and CAS severity, leukoaraiosis and infarcts all have been implicated in cognitive impairments. CAS severity was not only hypothesized to directly impede specific cognitive domains, but also transmit its effects indirectly to cognitive function through ipsilateral infarcts as well as periventricular leukoaraiosis (PVL) and deep white matter leukoaraiosis (DWML). We aimed to delineate the contributions of leukoaraiosis, infarcts and CAS to different specific cognitive domains.

**Materials and methods:**

One hundred and sixty one participants with unilateral CAS (>50%) on the left (*n* = 85) or right (*n* = 76) side and 65 volunteers without significant CAS (<50%) were recruited. The PVL, DWML, and infarct severity were visually rated on MRI. A comprehensive cognitive battery was administered and standardized based on age norms. Correlation and mediation analyses were adopted to examine the direct and indirect influence of CAS, leukoaraiosis, and infarct on specific cognitive domains with adjustment for education, hypertension, diabetes mellitus, and hyperlipidemia.

**Results:**

Carotid artery stenosis severity was associated with ipsilateral leukoaraiosis and infarct. Left CAS had direct effects on most cognitive domains, except for visual memory and constructional ability, and transmitted its indirect effects on all cognitive domains through ipsilateral PVL, and on constructional ability and psychomotor through infarcts. Right CAS only had negative direct effects on visual memory, psychomotor, design fluency and color processing speed, and transmitted its indirect effects on visual memory, word and color processing speed through ipsilateral infarcts. The trends of direct and indirect cognitive effects remained similar after covariate adjustment.

**Conclusion:**

Left and right CAS would predominantly lead to verbal and non-verbal cognitive impairment respectively, and such effects could be mediated through CAS-related leukoaraiosis and infarct. Given that cognition is subject to heterogeneous pathologies, the exact relationships between markers of large and small vessel diseases and their composite prognostic effects on cognition requires further investigation.

## Introduction

Carotid artery stenosis (CAS) is one of the major risk factors for stroke (Howard et al., 2021), and it can also lead to remarkable cognitive impairments even in patients with asymptomatic CAS (Chang et al., 2013). The neurocognitive changes in CAS patients are usually attributed to large vessel disease (LVD)-related chronic cerebral hypoperfusion and thrombotic emboli (Wallin et al., 2018). The direct associations between cerebral hypoperfusion and cognitive impairments in patients with CAS have been demonstrated by different imaging markers, including Doppler-based breath holding index (Balestrini et al., 2013), arterial spin labeling (Schröder et al., 2019) from MRI perfusion, and disrupted neural connectivity on functional MRI (Chang et al., 2016; Huang et al., 2018). Furthermore, left and right CAS are more related to verbal and non-verbal cognitive impairment as the lateralization effect (Huang et al., 2017). However, whether restoration of cerebral perfusion by carotid revascularization procedure is beneficial to cognition is inconclusive (Plessers et al., 2014).

On the other hand, leukoaraiosis and lacunar infarcts are manifestations of small vessel disease (SVD), and they have also been commonly observed in patients with CAS (Kandiah et al., 2014; Pini et al., 2020). Furthermore, the extent of leukoaraiosis is associated with CAS severity (Saba et al., 2009). Irrespective of their pathogeneses, leukoaraiosis (Appelman et al., 2010; Chowdhury et al., 2011) and infarcts (Saczynski et al., 2009) alone can independently contribute to cognitive impairments in individuals with advancing ages and various kinds of neurological conditions (Gorelick et al., 2011; Chui and Ramirez Gomez, 2017). As the origins of neurocognitive changes in CAS patients are multifactorial and inter-related, it is intrinsically complicated to investigate the relationship between cerebrovascular markers and cognition in CAS patients.

In literature, most studies investigating cognitive functions in patients with CAS have been based on group comparison designs to demonstrate cognitive sequalae from certain pathologies, but such approaches may not convey enough information regarding how CAS exerts its effect on cognition through the underlying LVD and SVD pathologies (Chang et al., 2013; Lal et al., 2017; Ihle-Hansen et al., 2021; Paraskevas et al., 2021). We reckoned to delineate these underlying pathologies on specific cognitive domains may have important implications for evaluating the treatment outcomes in patients with CAS. In this study, we proposed exploring the possible mediating roles of leukoaraiosis and infarcts in the associations between CAS and cognition might help to clarify these issues.

## Materials and methods

### Participants

A total of 161 participants with CAS (147 males, 14 females) attended for the outpatient clinics at the Department of Neurology, Linkou Chang Gung Memorial Hospital participated in this study. Their mean age was 65.7 years (SD = 8.5), ranging from 40 to 86 years old. They were recruited based on the following inclusion criteria, (a) unilateral internal carotid artery stenosis (either the left or right side of CAS > 50%); (b) the score of the Mini Mental State Examination (MMSE) ≥ 20; (c) the score for the Clinical Dementia Rating Scale (CDR) < 1; (d) right-hand dominance; (e) the scores on the National Institutes of Health Stroke Scale (NIHSS) ≤ 8; (f) the score for the Barthel Index ≥ 80; (g) the score for the Modified Rankin Scale ≤ 3. The exclusion criteria were: (a) stroke within the past 3 months at recruitment; (b) a history of psychiatric illness; (c) undergoing the coronary or peripheral arterial surgeries in the past 30 days at recruitment; (d) a history of traumatic head injury; (e) persistent moderate to severe dysphasia, which was defined as a score of > 1 point of the language item of NIHSS. According to the severity of left or right internal carotid artery stenosis, these participants were allocated to the left group (*n* = 85, stenosis of the left carotid artery > 50% and the right carotid artery < 50%), the right group (*n* = 76, stenosis of the left carotid artery < 50% and the right carotid artery > 50%).

In addition, 65 age- and gender-matched volunteers (54 males, 11 females) with stenosis of the left and right carotid arteries < 50% were recruited as the non-CAS group by advertisements placed around the hospital. Their mean age was 64.5 years (SD = 6.3), ranging from 54 to 81 years old. The inclusion criteria were as follows: (a) no history of neuropsychiatric disorders or head injuries; (b) the internal carotid artery stenosis on both sides were less than 50%. The reasons for including participants with no or milder CAS in this study were to substantiate the notion that cognitive impairments can be produced by severe CAS, and to increase the range of the grade of CAS as the predictor in mediation analyses.

The study protocol and procedure for obtaining informed consent were complied with the Helsinki Declaration and were approved by the Institutional Review Board of Chang Gung Memorial Hospital (IRB No. 201601092B0, 103-7584A, and 201601675A0). All participants signed the written informed consent.

### Imaging investigations

The grade of CAS in clinical participants was determined by digital subtraction angiography (DSA) according to the criteria proposed by the North American Symptomatic Carotid Endarterectomy Trial (NASCET) Collaborators (North American Symptomatic Carotid Endarterectomy Trial Collaborators, Barnett et al., 1991). The grade of CAS of control group was determined by brain magnetic resonance angiography or color-coded carotid duplex if the DSA was not available.

Brain MRI was performed within 1 month after enrollment for both CAS and non-CAS groups, and the method of MRI evaluation has been described in previous studies (Huang et al., 2018). In brief, anatomical MRI was obtained at a 1.5- or 3.0-Tesla scanner with 5-mm slice thickness and 0.5-mm inter-slice gap for all sequences, including axial T1-weighted, fluid-attenuated inversion recovery (FLAIR). The severity of infarcts and leukoaraiosis on each hemisphere was visually rated by two neurologists. The severity of infarcts in both hemispheres was converted into a four-point scale, rated as 0 = no lesion, 1 = one focal lesion (≥ 5 mm), 2 = more than one focal lesions, and 3 = confluent lesions (Lee et al., 2017). The severity of periventricular leukoaraiosis (PVL) and deep white matter leukoaraiosis (DWML) was assessed by Fazekas scale on FLAIR sequence (Fazekas et al., 1987). The severity of PVL was defined as 0 = absence, 1 = “caps or pencil-thin lining,” 2 = smooth “halo,” 3 = irregular leukoaraiosis extending into the deep white matter, whereas, the severity of DWML was defined as 0 = absence, 1 = punctuate foci, 2 = beginning confluence of foci, 3 = large confluent areas.

### Cognitive tests

#### Raw scores

A battery of comprehensive cognitive tests was administered in this study. These tests were chosen because our previous studies have shown they were sensitive to the cognitive deficits in patients with carotid stenosis (Huang et al., 2018, 2022). The CDR and MMSE were administered for general cognitive function evaluation. A Taiwanese adaptation of the California Verbal Learning Test-2 (Delis et al., 2000) and the Brief Visual Memory Test-Revised (BVMT-R) (Benedict, 1997) were administered on all participants to test verbal and visual memory, respectively. The Position Discrimination (PD) and Number Location (NL) subtests of the Visual Objection and Spatial Perception Battery (VOSP) (Warrington and James, 1991) were administered for testing the visuospatial functions. The constructional ability was examined by the Benton 3-Dimensional Construction Praxis Test (B3DCP) (Benton et al., 1994). The Purdue Pegboard Test (Tiffin, 1968) was used to measure the psychomotor function for both hands. The Color Trail Test (D’Elia et al., 1996) and the Category Fluency Test and Design Fluency Test of the Delis-Kaplan Executive System (Delis et al., 2001) were used to tap the verbal and non-verbal aspects of the executive functions. The mean reaction times of computerized Stroop word/color interference tests were used as the measures of processing speed as described in our previous study (Huang et al., 2022). The interval between the imaging investigations and cognitive assessment was less than 1 month for each participant.

#### T-score transformation

As more than 20 neuropsychological measures were recorded, a scale-reduction procedure was applied to minimize the potential redundancy of multiple comparisons (Huang et al., 2022). All raw testing scores for each participant were transformed to T-score (Mean = 50, SD = 10) based on the local corresponding age norms. In addition, we adopted a data-driven approach to carry out a principal component analysis (PCA) on all individually derived T-scores from approximately 40% (*n* = 94) of randomly selected participants in this study to extract the latent components corresponding to each cognitive measure. Seven components were extracted from this dataset by Varimax rotation, which accounted for > 72% of the total variance. The factor loadings for the cognitive measures corresponding to these components are summarized in Supplementary Table A. The T-scores of the cognitive measures within each component were averaged to derive the mean T-score for each specific cognitive domain.

### Data analysis

The demographic, clinical and cognitive data for each group were summarized by descriptive statistics. Depending on the scale of each measure, group comparisons were conducted by χ^2^ tests or one-way analysis of variance (ANOVA) where appropriate. Bonferroni corrections were applied where appropriate for the *post hoc* comparisons between each group.

#### Mediation analyses

**FIGURE 1:**
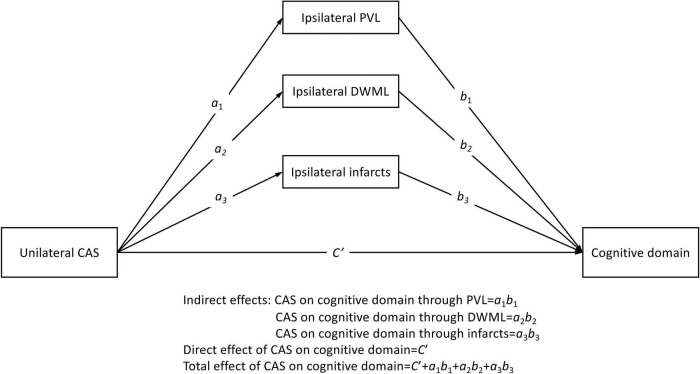
Conceptual parallel mediation model. Letters *a*_*i*_ are regression coefficients of the predictor variable in predicting three parallel mediators; and letters *b*_*i*_ and *C_*i*_’* are the regression coefficients of mediators and predictors in predicting outcome variables, respectively (*i* = 1 to 3). The error term in each prediction is not shown. CAS, carotid artery stenosis; PVL, periventricular leukoaraiosis; DWML, deep white matter leukoaraiosis.

We adopted the PROCESS macro for SPSS Version 4 developed by Hayes (2022) to carry out a series of mediation analyses. As being shown in Figure 1, grade of unilateral CAS (%CAS) was used as the predictor variable for the direct effect evaluation, and the ipsilateral PVL, DWML and infarcts severity were the three parallel mediators for the indirect effect evaluation. The outcome variable was the mean T-score for each specific cognitive domain. We discretely examined the associations between CAS in either side (left vs. right) and each specific cognitive domain by utilizing a model template [PROCESS Model 4, Hayes (2022)]. Since the sampling indirect effects of CAS on specific cognitive domains were unlikely to be normally distributed, a large number of samples (*n* = 5000) of data were taken to derive the empirically generated 95% bootstrap intervals of the sampling distributions of the indirect effects (Hayes, 2022). In addition, years of education and vascular risk factors were used as covariates in the mediation analysis models to examine their effects on each cognitive domain. Statistical significance would be claimed when the direct, indirect or total effects (i.e., sum of direct and indirect effects) of unilateral CAS severity on each specific cognitive domain by the absence of zero within the confidence intervals.

## Results

### Demographic and clinical data

Table 1 shows the demographic and clinical data for all groups. The mean ages and gender ratios did not significantly differ between groups (ps > 0.099). CAS patients had more prominent carotid stenosis severity and higher frequency of hypertension, hyperlipidemia, diabetic mellitus, coronary artery diseases and gouts than the non-CAS group, while the non-CAS group had higher education (ps < 0.019). The difference or proportion of the above demographic and clinical conditions did not significantly differ between CAS groups. The CAS groups tended to present with higher leukoaraiosis and infarct severity than the non-CAS group and the frequency distributions of PVL, DWML, and infarct severity in the left and right cerebral hemispheres among groups were statistically significant (ps < 0.05, see Supplementary Table B). In addition, the hemispheric PVL, DWML, and infarct severity tended to be more strongly correlated with the ipsilateral CAS severity than the contralateral CAS severity (Table 2).

**TABLE 1:** Demographic and medical data for all groups.

	Group				
	Non-CAS	Left	Right		
M ± SD [95%CI]	*n* = 65	*n* = 85	*n* = 76	*F*	*p*
Age, years	64.7 ± 6.3 [63.1, 66.3]	66.7 ± 8.3 [64.9, 68.5]	64.6 ± 8.5 [62.7, 66.6]	1.71	0.18
Education, years	11.2 ± 3.5 [10.3, 12.0]	9.0 ± 3.4 [8.3, 9.8]*	9.5 ± 3.8 [8.6, 10.4]*	6.87	0.001
Lt-CAS,%	10.1 ± 11.6 [7.2, 12.9]	80.1 ± 15.6 [76.7, 83.5]*	22.1 ± 18.3 [17.9, 26.8]*^†^	451.43	<0.001
Rt-CAS,%	8.0 ± 11.0 [5.3, 10.8]	21.0 ± 17.3 [17.3, 24.7]*	82.6 ± 14.0 [79.4, 85.8]*^†^	549.44	<0.001
***n* (%)**				**χ^2^**	***p***
Male	54 (83)	80 (94)	67 (88)	4.64	0.099
Hypertension	21 (32)	63 (74)*	62 (82)*	42.59	<0.001
Hyperlipidemia	25 (39)	51 (60)*	54 (71)*	15.57	<0.001
Diabetic mellitus	7 (11)	26 (31)*	29 (38)*	13.88	<0.001
CAD	3 (5)	20 (24)*	21 (28)*	13.34	0.001
Gout	4 (6)	21 (25)*	18 (24)*	9.97	0.002

*N* = 226. CI, confidence interval; Lt, left; Rt, right; CAS, carotid artery stenosis; CAD, coronary artery disease; PVL, periventricular leukoaraiosis; DWML, deep white matter leukoaraiosis.

*p < 0.05 compared with the control group.

^†^*p* < 0.05 compared with the left group.

**TABLE 2:** Correlation between age, education, and vascular pathologies of the left and right sides.

	1	2	3	4	5	6	7	8	9	M	SD
1. Age, years	—									65.4	7.9
2. Edu, years	−0.30**	—								9.8	3.7
3. Lt-CAS,%	0.15*	−0.22**	—							40.5	34.8
4. Rt-CAS,%	−0.03	−0.12	−0.01	—						38.0	35.4
5. Lt-PVL*^a^*	0.31**	−0.32**	0.25**	0.16*	—					—	—
6. Rt-PVL*^a^*	0.30**	−0.23**	0.11	0.10	0.78**	—				—	—
7. Lt-DWML*^a^*	0.12	−0.10	0.21**	0.02	0.36**	0.37**	—			—	—
8. Rt-DWML*^a^*	0.14*	−0.13	0.05	0.22**	0.42**	0.49**	0.64**	—		—	—
9. Lt-infarct*^a^*	0.15*	−0.08	0.34**	0.10	0.28**	0.23**	0.39**	0.24**	—	—	—
10. Rt-infarct*^a^*	0.06	−0.15*	0.04	0.38**	0.30**	0.24**	0.17*	0.32**	0.36**	—	—

*N* = 226. CAS, carotid artery stenosis; DWML, deep white matter leukoaraiosis; Edu, education; Lt, left; PVL, periventricular leukoaraiosis; Rt, right.

^a^Based on Spearman ρ correlation analysis.

*p < 0.05; **p < 0.01.

### Cognitive function comparisons

The mean (± SD) raw scores of the cognitive tests in this study are shown in Supplementary Table C. One-way ANOVA revealed that except for the NL subtest of the VOSP (*p* = 0.064), the effect of group was statistically significant on each measure (Fs > 3.59, ps < 0.03). *Post hoc* comparisons between groups indicated that the mean raw scores of nearly all cognitive measures for the left groups were significantly lower than those for the non-CAS group (ps < 0.05), except for the B3DCPT and VOSP scores. Likewise, most of the cognitive measures for the right group were also significantly inferior to those of the non-CAS group (ps < 0.05), but not the NL subtest of the VOSP. The differences in the mean raw scores of all cognitive measures between the two clinical groups were not significant (ps > 0.08).

### Correlation and mediation analyses between cognition and vascular markers

The correlations of mean T-scores of specific cognitive domains with demographic data, CAS severity, leukoaraiosis and infarcts are demonstrated in Table 3. Overall, the verbal and visual memory, psychomotor function, design fluency and color processing speed were correlated with most of the vascular markers. However, the construction ability was not correlated with bilateral CAS severity, hypertension, hyperlipidemia or Rt-Infarct, neither was word processing speed correlated with Rt-CAS, hyperlipidemia, diabetes mellitus, or Lt-DWML.

**TABLE 3:** Correlation of cognition with demographic data and severity of carotid artery stenosis (CAS), leukoaraiosis, and infarct for the total sample.

	Verbal memory	Visual memory	Construction	Psychomotor	Design-FL	Word-PS	Color-PS
Edu, years	0.38**	0.38**	0.31**	0.18**	0.38**	0.32**	0.26**
Hypertension	−0.21**	−0.25**	−0.12	−0.29**	−0.22**	−0.27**	−0.26**
Hyperlipidemia	−0.18**	−0.05	−0.12	−0.15*	−0.16*	0.03	0.01
Diabetic mellitus	−0.11	−0.18**	−0.18**	−0.21**	−0.18**	−0.12	−0.13
Lt-CAS, %	−0.36**	−0.18**	−0.08	−0.24**	−0.24**	−0.37**	−0.31**
Rt-CAS, %	−0.17**	−0.29**	−0.11	−0.27**	−0.25**	−0.12	−0.28**
Lt-PVL*^a^*	−0.24**	−0.29**	−0.31**	−0.32**	−0.33**	−0.36**	−0.37**
Rt-PVL*^a^*	−0.18*	−0.21**	−0.24**	−0.26**	−0.25**	−0.25**	−0.30**
Lt-DWML*^a^*	−0.17*	−0.18**	−0.22**	−0.12	−0.22**	−0.11	−0.15*
Rt-DWML*^a^*	−0.14*	−0.22**	−0.23**	−0.21**	−0.24**	−0.15*	−0.22**
Lt-Infarct*^a^*	−0.24**	−0.21**	−0.22**	−0.25**	−0.25**	−0.21**	−0.25**
Rt-Infarct*^a^*	−0.20**	−0.26**	−0.10	−0.22**	−0.28**	−0.26**	−0.30**
M ± SD	45.59 ± 11.20	43.01 ± 11.43	46.00 ± 13.24	41.56 ± 10.88	45.37 ± 8.99	43.75 ± 13.42	41.17 ± 14.67

*N* = 226. CAS, carotid artery stenosis; Design-FL, design fluency; DWML, deep white matter leukoaraiosis; Edu, years of education; Lt, left; PS, processing speed; PVL, periventricular leukoaraiosis; Rt, right.

^a^Based on Spearman ρ correlation analysis.

*p < 0.05; **p < 0.01.

**FIGURE 2:**
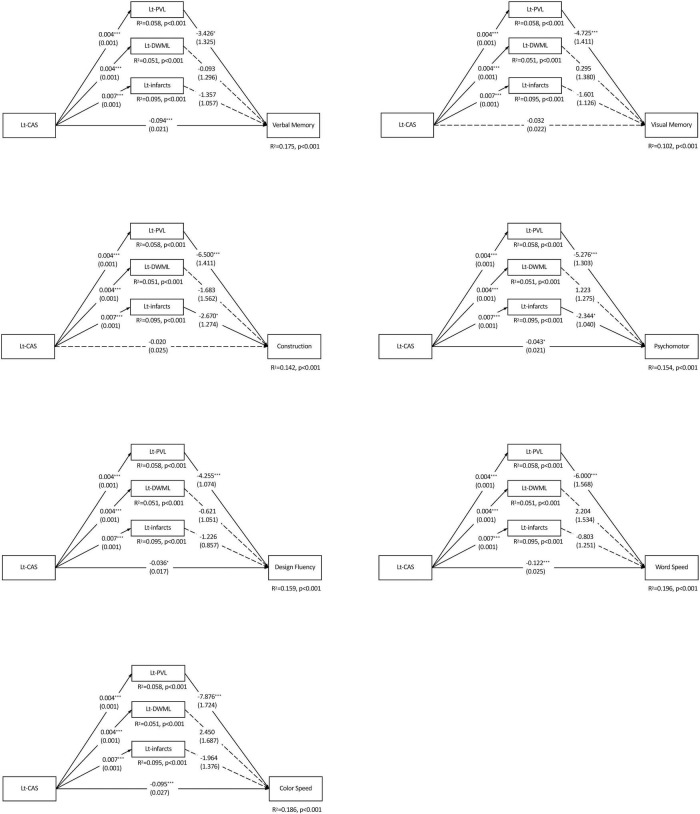
Parallel mediation models of each specific cognitive domain predicted by the left carotid artery stenosis and ipsilateral leukoaraiosis and infarcts. *N* = 226. The value in each path is the regression coefficient (standard error). Lt, left; CAS, carotid artery stenosis; PVL, periventricular leukoaraiosis; DWML, deep white matter leukoaraiosis; R^2^, coefficient of determination. *p < 0.05; **p < 0.01; ***p < 0.001.

Considering the inter-relations among CAS, leukoaraiosis, infarcts and cognition, mediation analyses were adopted to delineate the influence of each vascular component on cognitive domains. As shown in Figure 2, Lt-CAS positively predicted the ipsilateral PVL, DWML and infarcts severity (ps < 0.001), accounting for more than 10% (from 10.2 to 19.6%) of all variances in specific cognitive domains. Lt-CAS directly predicted most of the cognitive domains, except for visual memory and constructional ability, and its indirect effects on all cognitive domains through ipsilateral PVL, as well as on constructional ability and psychomotor domains through Lt-infarct, were significant. After adjusting for education, hypertension, hyperlipidemia and diabetes mellitus, some of the effects were diminished, including (1) Lt-CAS direct effects on psychomotor and design fluency, (2) Lt-PVL indirect effects on verbal and visual memory, and (3) Lt-infarct indirect effects on constructional and psychomotor domains (see Table 4).

**TABLE 4:** Effects of unilateral carotid artery stenosis on specific cognitive domains in parallel mediation analyses.

		Predictor (Left carotid artery stenosis, %)				Predictor (Right carotid artery stenosis, %)			
		Unadjusted		Adjusted		Unadjusted		Adjusted	
Domains		Effect (SE)	95%CI	Effect (SE)	95%CI	Effect (SE)	95%CI	Effect (SE)	95%CI
Verbal memory	*Total*	−0.117 (0.020)	**[−0.156, −0.078]**	**−0.085 (0.020)**	**[−0.123, −0.046]**	**−0.053 (0.021)**	**[−0.094, −0.012]**	−0.018 (0.021)	[−0.059, 0.022]
	*Direct*	**−0.094 (0.021)**	**[−0.136, −0.053]**	**−0.074 (0.021)**	**[−0.115, −0.034]**	−0.034 (0.022)	[−0.078, 0.010]	−0.0001 (0.003)	[−0.054, 0.032]
	*Indirect*								
	PVL	**−0.0135 (0.007)**	[**−0.028**, **−0.002**]	−0.004 (0.004)	[−0.014, 0.002]	−0.005 (0.005)	[−0.016, 0.002]	−0.0001 (0.003)	[−0.006, 0.005]
	DWML	−0.0004 (0.005)	[−0.011, 0.010]	0.001 (0.004)	[−0.009, 0.010]	0.001 (0.006)	[−0.010, 0.014]	0.002 (0.004)	[−0.005, 0.012]
	Infarct	−0.009 (0.007)	[−0.024, 0.004]	−0.007 (0.006)	[−0.020, 0.004]	−0.014 (0.009)	[−0.032, 0.002]	−0.009 (0.008)	[−0.025, 0.005]
		R^2^ = 0.175, *F* = 11.726***		R^2^ = 0.264, *F* = 9.743***		R^2^ = 0.077, *F* = 4.630**		R^2^ = 0.205, *F* = 7.002***	
Visual memory	*Total*	**−0.060 (0.022)**	**[−0.103, −0.018]**	−0.020 (0.021)	[−0.061, 0.021]	**−0.094 (0.021)**	**[−0.135, −0.053]**	**−0.069 (0.020)**	**[−0.109, −0.029]**
	*Direct*	−0.032 (0.022)	[−0.076, 0.012]	−0.009 (0.021)	[−0.051, 0.034]	**−0.069 (0.022)**	**[−0.112, −0.026]**	**−0.057 (0.021)**	**[−0.099, −0.015]**
	*Indirect*								
	PVL	**−0.019 (0.007)**	**[−0.035, −0.006]**	−0.005 (0.004)	[−0.016, 0.001]	−0.005 (0.004)	[−0.013, 0.002]	−0.0001 (0.002)	[−0.004, 0.004]
	DWML	0.001 (0.006)	[−0.010, 0.013]	0.001 (0.001)	[−0.010, 0.011]	−0.003 (0.006)	[−0.015, 0.009]	−0.003 (0.004)	[−0.012, 0.004]
	Infarct	−0.010 (0.007)	[−0.026, 0.003]	−0.005 (0.006)	[−0.020, 0.006]	**−0.018 (0.009)**	**[−0.037, −0.006]**	−0.010 (0.008)	[−0.026, 0.005]
		R^2^ = 0.102, *F* = 6.255***		R^2^ = 0.225, *F* = 7.854***		R^2^ = 0.143, *F* = 9.184***		R^2^ = 0.263, *F* = 9.668***	
Construction	*Total*	−0.030 (0.025)	[−0.080, 0.020]	0.008 (0.025)	[−0.042, 0.057]	−0.042 (0.025)	[−0.091, 0.007]	−0.016 (0.025)	[−0.066, 0.034]
	*Direct*	0.020 (0.025)	[−0.030, 0.070]	0.039 (0.025)	[−0.011, 0.088]	−0.025 (0.026)	[−0.076, 0.027]	−0.014 (0.026)	[−0.065, 0.038]
	*Indirect*								
	PVL	**−0.026 (0.009)**	**[−0.045, −0.011]**	**−0.012 (0.007)**	[**−0.026**, **−0.001**]	−0.008 (0.006)	[−0.023, 0.002]	−0.0002 (0.005)	[−0.010, 0.009]
	DWML	−0.007 (0.006)	[−0.021, 0.004]	−0.006 (0.006)	[−0.019, 0.003]	−0.008 (0.007)	[−0.024, 0.004]	−0.005 (0.006)	[−0.018, 0.003]
	Infarct	**−0.017 (0.009)**	**[−0.037, −0.001]**	−0.013 (0.008)	[−0.031, 0.0003]	−0.001 (0.011)	[−0.024, 0.020]	0.003 (0.009)	[−0.016, 0.021]
		R^2^ = 0.142, *F* = 9.178***		R^2^ = 0.210, *F* = 7.225***		R^2^ = 0.093, *F* = 5.651***		R^2^ = 0.173, *F* = 5.680***	
Psychomotor	*Total*	**−0.075 (0.020)**	**[−0.115, −0.035]**	**−0.047 (0.020)**	**[−0.087, −0.006]**	**−0.083 (0.020)**	**[−0.122, −0.043]**	**−0.054 (0.021)**	**[−0.095, −0.014]**
	*Direct*	**−0.043 (0.021)**	**[−0.084, −0.003]**	−0.31 (0.021)	[−0.072, 0.010]	**−0.063 (0.021)**	**[−0.104, −0.023]**	**−0.048 (0.021)**	**[−0.089, −0.006]**
	*Indirect*								
	PVL	**−0.021 (0.007)**	**[−0.036, −0.008]**	**−0.011 (0.006)**	[**−0.023**, **−0.001**]	−0.007 (0.006)	[−0.020, 0.002]	−0.0002 (0.004)	[−0.010, 0.008]
	DWML	0.005 (0.005)	[−0.006, 0.016]	0.004 (0.005)	[−0.005, 0.015]	−0.003 (0.006)	[−0.016, 0.008]	−0.002 (0.004)	[−0.011, 0.006]
	Infarct	**−0.015 (0.008)**	**[−0.032, −0.002]**	−0.009 (0.007)	[−0.024, 0.003]	−0.009 (0.009)	[−0.028, 0.009]	−0.005 (0.008)	[−0.021, 0.011]
		R^2^ = 0.154, F = 10.052***		R^2^ = 0.202, F = 6.869***		R^2^ = 0.159, F = 10.472***		R^2^ = 0.205, F = 6.989***	
Design fluency	*Total*	**−0.063 (0.017)**	**[−0.096, −0.030]**	**−0.033 (0.016)**	**[−0.065, −0.001]**	**−0.064 (0.016)**	**[−0.096, −0.031]**	**−0.039 (0.016)**	**[−0.071, −0.007]**
	*Direct*	**−0.036 (0.017)**	**[−0.069, −0.002]**	−0.019 (0.017)	[−0.051, 0.014]	**−0.043 (0.017)**	**[−0.076, −0.009]**	−0.030 (0.017)	[−0.063, 0.003]
	*Indirect*								
	PVL	**−0.017 (0.006)**	**[−0.030, −0.007]**	**−0.007 (0.004)**	[**−0.016**, **−0.0002**]	−0.005 (0.004)	[−0.014, 0.002]	−0.0001 (0.003)	[−0.006, 0.005]
	DWML	−0.002 (0.004)	[−0.012, 0.005]	−0.002 (0.004)	[−0.011, 0.005]	−0.003 (0.005)	[−0.014, 0.005]	−0.002 (0.003)	[−0.010, 0.004]
	Infarct	−0.008 (0.005)	[−0.018, 0.003]	−0.005 (0.004)	[−0.014, 0.003]	−0.012 (0.007)	[−0.026, 0.001]	−0.007 (0.006)	[−0.020, 0.004]
		R^2^ = 0.159, *F* = 10.462***		R^2^ = 0.252, *F* = 9.150***		R^2^ = 0.154, *F* = 10.060***		R^2^ = 0.257, *F* = 9.383***	
Word PS	*Total*	**−0.143 (0.024)**	**[−0.190, −0.096]**	**−0.110 (0.024)**	**[−0.157, −0.064]**	−0.045 (0.025)	[−0.095, 0.004]	−0.011 (0.025)	[−0.060, 0.038]
	*Direct*	**−0.122 (0.025)**	**[−0.171, −0.073]**	**−0.105 (0.024)**	**[−0.153, −0.056]**	−0.013 (0.026)	[−0.064, 0.039]	−0.007 (0.026)	[−0.044, 0.058]
	*Indirect*								
	PVL	**−0.024 (0.008)**	**[−0.041, −0.010]**	**−0.009 (0.005)**	[**−0.021**, **−0.0004**]	−0.008 (0.006)	[−0.022, 0.002]	−0.0002 (0.003)	[−0.007, 0.007]
	DWML	0.008 (0.006)	[−0.004, 0.021]	0.004 (0.005)	[−0.006, 0.016]	0.008 (0.008)	[−0.005, 0.025]	0.004 (0.005)	[−0.004, 0.017]
	Infarct	−0.005 (0.009)	[−0.024, 0.011]	−0.001 (0.007)	[−0.017, 0.013]	**−0.034 (0.012)**	**[−0.058, −0.011]**	**−0.022 (0.010)**	**[−0.043, −0.004]**
		R^2^ = 0.196, *F* = 13.438***		R^2^ = 0.270, *F* = 10.010***		R^2^ = 0.104, *F* = 6.441***		R^2^ = 0.213, *F* = 7.334***	
Color PS	*Total*	**−0.129 (0.027)**	**[−0.182, −0.076]**	**−0.096 (0.027)**	**[−0.149, −0.042]**	**−0.116 (0.027)**	**[−0.169, −0.064]**	**−0.091 (0.027)**	**[−0.144, −0.037]**
	*Direct*	**−0.095 (0.027)**	**[−0.149, −0.041]**	**−0.080 (0.028)**	**[−0.134, −0.026]**	**−0.080 (0.027)**	**[−0.134, −0.026]**	**−0.070 (0.028)**	**[−0.125, −0.015]**
	*Indirect*								
	PVL	**−0.031 (0.010)**	**[−0.054, −0.013]**	**−0.015 (0.008)**	[**−0.033**, **−0.001**]	−0.010 (0.007)	[−0.026, 0.003]	−0.0003 (0.005)	[−0.012, 0.011]
	DWML	0.010 (0.007)	[−0.003, 0.024]	0.006 (0.006)	[−0.004, 0.019]	0.005 (0.008)	[−0.008, 0.023]	0.002 (0.005)	[−0.007, 0.014]
	Infarct	−0.013 (0.010)	[−0.033, 0.005]	−0.007 (0.008)	[−0.025, 0.008]	**−0.032 (0.013)**	**[−0.061, −0.007]**	**−0.023 (0.012)**	**[−0.047, −0.001]**
		R^2^ = 0.186, *F* = 12.580***		R^2^ = 0.227, *F* = 7.978***		R^2^ = 0.182, *F* = 12.321***		R^2^ = 0.232, *F* = 8.179***	

*N* = 226. Values in adjusted columns represent the estimated effects, standard errors, and 95% confidence intervals by using years of education, hypertension, hyperlipidemia and diabetic mellitus as the covariates. Total effect of carotid artery stenosis on each specific cognitive domain is the sum of direct and indirect effects, which is equal to the estimate of regressing each specific cognitive domain on carotid artery stenosis on either side, respectively. The direct effect of carotid artery stenosis on each specific cognitive domain was estimated while the parallel mediators being remained constant. PVL, periventricular leukoaraiosis; DWML, deep while matter leukoaraiosis; SE, standard error; CI, confidence interval; PS, processing speed; R^2^, coefficients of determination of the parallel mediation models. Significant estimated effects of predictors (carotid artery stenosis on each side, respectively) are presented in bold typeface.

**p < 0.01; ***p < 0.001.

**FIGURE 3:**
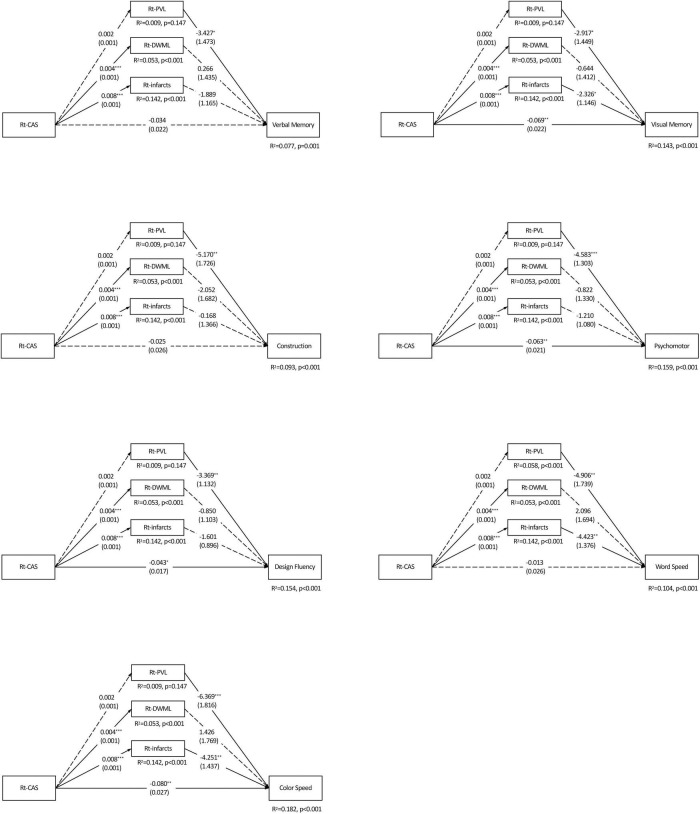
Parallel mediation models of each specific cognitive domain predicted by the right carotid artery stenosis and ipsilateral leukoaraiosis and infarcts. *N* = 226. The value in each path is the regression coefficient (standard error). Rt, right; CAS, carotid artery stenosis; PVL, periventricular leukoaraiosis; DWML, deep white matter leukoaraiosis; R^2^, coefficient of determination. *p < 0.05; **p < 0.01; ***p < 0.001. Significant estimated effects of predictors (carotid artery stenosis on each side, respectively) are presented in bold typeface.

Figure 3 shows Rt-CAS only positively predicted the severity of ipsilateral DWML and infarcts (ps < 0.001), respectively, but not ipsilateral PVL (p = 0.957), which accounted for more than 7% (from 7.7 to 18.2%) of all variances of the specific cognitive domains. As shown in Table 4, Rt-CAS directly predicted non-verbal domains, including visual memory, psychomotor and design fluency, but not the other domains; and its indirect effects were only significant on visual memory, word and color processing speed through ipsilateral infarcts. After adjustment of education and vascular risk factors, only the direct effects of Rt-CAS on design fluency and its indirect effects on visual memory through Rt-infarct were abolished.

## Discussion

In this study, our results further substantiated the general notion that CAS can lead to cognitive impairments through multiple mechanisms. In good agreement with previous findings, we found the CAS severity was correlated with its ipsilateral PVL, DWML, and infarct severity, but not the contralateral ones, suggesting the effects of CAS on leukoaraiosis and infarcts might be lateralized (Baradaran et al., 2017; Benli et al., 2021). In correlation analyses, either left or right CAS severity were correlated with most cognitive tests. The lateralization effect on cognition from unilateral carotid stenosis was further demonstrated by mediation analyses in that left and right CAS were associated with verbal and non-verbal cognitive functions, respectively. Moreover, we observed the cognitive sequalae directly predicted by CAS severity, and indirectly through left periventricular leukoaraiosis and ipsilateral infarcts, suggesting CAS-associated leukoaraiosis and infarcts might play a mediating role in impairments in different specific cognitive domains.

### Indirect effects of carotid artery stenosis

#### Leukoaraiosis

Leukoaraiosis is associated with carotid atherosclerosis, and it has been recognized as a risk for cognitive impairment in both neurodegenerative and vascular diseases (Saba et al., 2009; Price et al., 2012; Lucatelli et al., 2016; Sam et al., 2016). In this study, the negative indirect effects of Lt-CAS through Lt-PVL were prevalent and significant on most cognitive domains even after adjustment of education and vascular risk factors. The findings were in agreement with an earlier review suggesting that PVL has negative impacts on cognitive abilities (Bolandzadeh et al., 2012; Wiggins et al., 2019). However, there was general lack of indirect effects of Rt-CAS through Rt-PVL and Rt-DWML on any specific cognitive domains. One possible reason was that the ipsilateral PVL severity in Lt-CAS happened to be more severe than that of Rt-CAS (Supplementary Table B), and some have argued that cognitive impairments can be attributed to leukoaraiosis only when the leukoaraiosis severity exceeds certain thresholds (Zeng et al., 2019).

Leukoaraiosis has great diversity in spatial distribution and signal intensity, and various leukoaraiosis quantification methods have been developed (Pantoni et al., 2002; Rost et al., 2014). Our study applied Fazekas scale to evaluate leukoaraiosis severity, as it is one of the well-established visual rating scales and also has good agreement with other visual scoring systems (Pantoni et al., 2002). Visual rating scales can be easily and reliably administered in clinical practice, and they have been proved to provide valuable prognostic information (Verdelho et al., 2012; Rudilosso et al., 2019). However, visual rating scales typically allow for a small number of ordinal ratings, and they may have limited sensitivity to subtle changes as compared to voxel-based volumetric quantification (Lambert et al., 2016; Garnier-Crussard et al., 2022). Therefore, voxel-based volumetric quantification methods might be able to provide more delicate information as to leukoaraiosis evolution in response to medical and intervention treatment in future studies.

#### Infarcts

Similar to leukoaraiosis, the severity of infarcts was also relatively mild (medians = 0 for both sides) in this study. Despite the infarct severity was negatively associated with nearly all cognitive domains in simple correlation analyses, their cognitive effects were greatly diminished in the mediation models. Although the relationships between infarcts and cognitive performance have been well-documented (Sigurdsson et al., 2017; Weaver et al., 2021), it is possible the influences of infarcts on cognitive domains might be balanced out because more than 50% of all participants who were infarct-free in this study (Supplementary Table B).

### Direct effects of carotid artery stenosis

Many studies have shown grade of CAS is inversely associated with cognitive performance (Wang et al., 2016; Porcu et al., 2020). In fact, cognition is a generic term encompassing various specific intellectual abilities, which are not only vulnerable to heterogeneous brain pathologies, but also strongly associated with demographic and vascular risk factors (Cheng et al., 2020; Desideri and Bocale, 2021). Our study findings were in line with previous studies showing that Rt-CAS was more closely related to non-verbal functions, whereas Lt-CAS tended to associate with more pervasive cognitive domains (Huang et al., 2014, 2017, 2018). The lateralization cognitive effects of unilateral CAS remained after controlling for education and multiple vascular risk factors. On the other hand, CAS severity could only be deemed as a proxy indicator of cerebral chronic hypoperfusion as collateral blood supply from the circle of Willis or ophthalmic artery may ameliorate cerebral hemodynamics. Therefore, incorporation of perfusion information in the mediation models would further differentiate the cognitive effects from LVD and SVD pathologies in future studies.

### Limitations

This study might represent one of few attempts to bring multiple pathophysiological factors together for exploring the possible underlying mediators on the associations between CAS and cognition, yet it had several limitations. First, the major contributors (brain perfusion status and hemodynamics) to cognitive impairments in patients with CAS were not included for analyses. Although some participants with CAS had the perfusion data available in this study, inadequate sample size might render inclusion of perfusion data for mediation analyses inappropriate. Second, the non-CAS group was recruited to contrast the CAS effects on cognition. However, their educational attainments and other vascular risk factors were different from the CAS groups, which could only be taken into account by statistical adjustment. Third, even though the severity in leukoaraiosis and infarcts in the participants of this study were relatively mild, their mediating effects were still detectable in this study. As leukoaraiosis and infarct severity are diverse in CAS patients, generalization of the present results should be cautious. Moreover, the variances of individual specific cognition domains could be accounted for by the variables included in this study appeared modest. In fact, cognition can be influenced by many pathogeneses beyond the present selection. It is deemed necessary to have a comprehensive inclusion of potential risk factors for mediation analysis in future study.

In conclusion, unilateral CAS would aggravate ipsilateral leukoaraiosis and infarct severity. Left and right CAS would predominantly lead to verbal and non-verbal cognitive impairment respectively, and such effects could be mediated through CAS-related leukoaraiosis and infarct. Given that cognition is subject to heterogenous pathologies, the exact relationships between LVD and SVD markers and their composite prognostic effects on cognition require further investigation.

## Data availability statement

The datasets presented in this article are not readily available because the used consent does not allow for the public sharing of the data. Requests to access the datasets should be directed to M-YH, myho@mail.cgu.edu.tw.

## Ethics statement

The studies involving human participants were reviewed and approved by Institutional Review Board of Chang Gung Memorial Hospital. The patients/participants provided their written informed consent to participate in this study.

## Author contributions

K-LH wrote a portion of the manuscript, revised the initial draft, performed neurologic examinations, and took part in the data collection and analysis and scientific interpretation of data. T-YC, Y-JC, H-CW, and C-HL performed the data collection, neurologic examination, and scientific interpretation of data. Y-MW performed MRI analysis and had scientific interpretation of data. T-HL conceptualized the study design, took part in critical review of the manuscript, and edited the manuscript for content. M-YH conceptualized the study design, wrote the initial draft, conducted the cognitive evaluation, and carried out statistical analyses and critical review of the manuscript. All authors critically reviewed the manuscript and approved the final version for publication.
