# Supplementary material for: Mediating roles of leukoaraiosis and infarcts in the effects of unilateral carotid artery stenosis on cognition
Source: Front Aging Neurosci. 2022 Sep 29;14:972480. doi: 10.3389/fnagi.2022.972480 (PMC9559387; doi:10.3389/fnagi.2022.972480)
Supplement: Supplementary file 1 [file Data_Sheet_1.docx]

**Supplemental Table A** Cognitive Measures and Factor Loadings Corresponding to Specific Cognitive Domain

| Cognitive domains | Test Scales | Factor Loadings |
| --- | --- | --- |
| Verbal Memory | Immediate Recall of the CVLT-2  Delay Recall of the CVLT-2  Recognition *d’* of the CVLT-2 | 0.786  0.758  0.801 |
| Visual Memory | Immediate Recall of the BVMT-R  Delay Recall of the BVMT-R  Recognition *d’* of the BVMT-R  Position Discrimination of the VOSP | 0.591  0.631  0.648  0.603 |
| Construction | Benton 3-D Construction Praxis Test | 0.874 |
| Psychomotor | Dominant hand (right) of the PPT  Non-dominant hand (left) of the PPT  Number Location of the VOSP | 0.630  0.785  0.531 |
| Visual Executive | Filled Dot of the DFT  Empty Dot of the DFT  Switch Trial of the DFT | 0.827  0.806  0.645 |
| Word Processing Speed | Word congruent trial of the Stroop Test  Word neutral trial of the Stroop Test  Word incongruent trial of the Stroop Test | 0.833  0.875  0.866 |
| Color Processing Speed | Trial 1 of the Color Trail Test  Trial 2 of the Color Trail Test  Color congruent trial of the Stroop Test  Color neutral trial of the Stroop Test  Color incongruent trial of the Stroop Test | 0.680  0.653  0.708  0.727  0.826 |
| The factor loadings for cognitive measures were derived from Varimax rotation by using principal component analysis of the T scores of all measures on 94 participants. BVMT-R=Brief Visual Memory Test-Revised; CVLT-2=California Verbal Learning Test, 2^nd^ ed.; d’=discriminability; DFT=Design Fluency Test; PPT=Purdue Pegboard Test; VOSP=Visual Object and Space Perception Battery. | | |

**Supplemental Table B** Frequency Distributions of Leukoaraiosis and Infarct Severity among the control, left ICA and right ICA groups

|  |  | Left cerebral hemisphere | | | | | |  | Right cerebral hemisphere | | | | | |
| --- | --- | --- | --- | --- | --- | --- | --- | --- | --- | --- | --- | --- | --- | --- |
| Group |  | Control |  | Left ICA |  | Right ICA |  |  | Control |  | Left ICA |  | Right ICA |  |
| n |  | 65 |  | 85 |  | 76 |  |  | 65 |  | 85 |  | 76 |  |
| PVL, n(%) |  | Median=1, χ^2^=19.90, p<0.001 | | | | |  |  | Median=1, χ^2^=13.24, p=0.039 | | | | |  |
| =0 |  | 39 (60) |  | 26 (31) |  | 23 (30) |  |  | 30 (46) |  | 33 (39) |  | 22 (29) |  |
| =1 |  | 26 (40) |  | 52 (61) |  | 49 (65) |  |  | 35 (54) |  | 45 (53) |  | 51 (67) |  |
| =2 |  | 0 (0) |  | 7 (8) |  | 4 (5) |  |  | 0 (0) |  | 7 (8) |  | 2 (3) |  |
| =3 |  | 0 (0) |  | 0 (0) |  | 0 (0) |  |  | 0 (0) |  | 0 (0) |  | 1 (1) |  |
| DWML, n(%) |  | Median=1, χ^2^=10.27, p=0.036 | | | | |  |  | Median=1, χ^2^=12.80, p=0.046 | | | | |  |
| =0 |  | 30 (46) |  | 26 (31) |  | 27 (36) |  |  | 33 (51) |  | 39 (46) |  | 21 (28) |  |
| =1 |  | 34 (52) |  | 47 (55) |  | 44 (58) |  |  | 31 (47) |  | 40 (47) |  | 47 (62) |  |
| =2 |  | 1 (2) |  | 12 (14) |  | 5 (6) |  |  | 1 (2) |  | 6 (7) |  | 7 (9) |  |
| =3 |  | 0 (0) |  | 0 (0) |  | 0 (0) |  |  | 0 (0) |  | 0 (0) |  | 1 (1) |  |
| Infarcts, n (%) |  | Median=0, χ^2^=33.08, p<0.001 | | | | |  |  | Median=0, χ^2^=31.65, p<0.001 | | | | |  |
| =0 |  | 63 (96) |  | 47 (55) |  | 56 (74) |  |  | 63 (97) |  | 68 (80) |  | 44 (58) |  |
| =1 |  | 1 (2) |  | 18 (21) |  | 8 (10) |  |  | 2 (3) |  | 6 (7) |  | 12 (16) |  |
| =2 |  | 1 (2) |  | 20 (24) |  | 12 (16) |  |  | 0 (0) |  | 11 (13) |  | 20 (26) |  |
| N=226. Numerical values (0-3) represent the visually-rated severity in leukoaraiosis and infarcts, respectively. Lt=left; Rt=right; ICA=internal carotid artery group; CAS=carotid artery stenosis; DWML=deep white matter leukoaraiosis; PVL=periventricular leukoaraiosis. | | | | | | | | | | | | | | |

**Supplemental Table C** Mean (±SD) Raw Scores of the Neuropsychological Tests for All Groups

|  | Control |  | Left |  | Right |  | χ^2^ or F | p |
| --- | --- | --- | --- | --- | --- | --- | --- | --- |
| *n* | 65 |  | 85 |  | 76 |  |  |  |
| CDR=0.5*^a^*, *n* (%) | 15 (23) |  | 36 (42) |  | 39 (51) |  | 12.02 | 0.002 |
| MMSE | 27.54±1.61 |  | 26.58±2.54 |  | 26.49±2.77 |  | 4.08 | 0.018 |
| Est-RSPM | 33.87±4.47 |  | 30.67±6.13^*^ |  | 32.10±5.87 |  | 5.99 | 0.003 |
| *Verbal Memory* |  |  |  |  |  |  |  |  |
| CVLT-2 |  |  |  |  |  |  |  |  |
| Immediate Recall | 40.83±8.32 |  | 30.18±11.65^*^ |  | 33.16±11.70^*^ |  | 18.44 | <0.001 |
| Delay Recall | 8.71±3.16 |  | 5.65±3.87^*^ |  | 5.97±3.89^*^ |  | 14.56 | <0.001 |
| Recognition | 2.62±0.83 |  | 1.80±0.91^*^ |  | 1.91±0.92^*^ |  | 17.49 | <0.001 |
| *Visual Memory* |  |  |  |  |  |  |  |  |
| BVMT-R |  |  |  |  |  |  |  |  |
| Immediate Recall | 18.18±7.38 |  | 13.37±6.80^*^ |  | 13.13±8.04^*^ |  | 10.10 | <0.001 |
| Delay Recall | 8.15±3.09 |  | 5.49±3.35^*^ |  | 5.63±3.67^*^ |  | 13.41 | <0.001 |
| Recognition | 5.43±1.04 |  | 4.72±1.32^*^ |  | 4.18±1.72^*^ |  | 13.62 | <0.001 |
| VOSP-PD | 19.38±1.10 |  | 19.13±1.13 |  | 18.61±1.80^*^ |  | 5.03 | 0.007 |
| *Construction* |  |  |  |  |  |  |  |  |
| B3DCPT | 23.12±5.48 |  | 21.30±6.07 |  | 20.26±7.06^*^ |  | 3.59 | 0.029 |
| *Psychomotor* |  |  |  |  |  |  |  |  |
| PPT-Rt hand | 14.02±1.79 |  | 11.17±2.89^*^ |  | 11.58±1.95^*^ |  | 30.30 | <0.001 |
| PPT-Lt hand | 12.88±1.77 |  | 11.42±2.24^*^ |  | 10.58±2.73^*^ |  | 17.22 | <0.001 |
| VOSP-NL | 8.03±2.14 |  | 7.93±2.07 |  | 7.14±2.48 |  | 2.80 | 0.064 |
| *Design Fluency* |  |  |  |  |  |  |  |  |
| Design Fluency Test |  |  |  |  |  |  |  |  |
| Filled dot trial | 7.14±2.69 |  | 5.93±2.41^*^ |  | 5.75±3.22^*^ |  | 5.02 | 0.007 |
| Empty dot trial | 8.65±3.21 |  | 6.54±3.04^*^ |  | 6.25±3.19^*^ |  | 11.72 | <0.001 |
| Switch trial | 6.86±2.25 |  | 5.08±2.36^*^ |  | 5.17±2.81^*^ |  | 11.18 | <0.001 |
| *Word Processing Speed* | |  |  |  |  |  |  |  |
| Stroop Tests | |  |  |  |  |  |  |  |
| Word congruent, ms | 904.2±207.3 |  | 1180.2±379.5^*^ |  | 1097.8±288.8^*^ |  | 14.33 | <0.001 |
| Word neural, ms | 949.6±231.0 |  | 1229.3±397.5^*^ |  | 1153.9±336.0^*^ |  | 12.52 | <0.001 |
| Word incongruent, ms | 1046.3±246.3 |  | 1321.3±415.4^*^ |  | 1234.7±358.2^*^ |  | 10.67 | <0.001 |
| *Color Processing Speed* |  |  |  |  |  |  |  |  |
| Color Trail Test |  |  |  |  |  |  |  |  |
| Trial 1, s | 54.91±28.00 |  | 98.61±124.55^*^ |  | 93.06±47.20^*^ |  | 5.17 | 0.007 |
| Trial 2, s | 125.26±54.23 |  | 157.97±69.99^*^ |  | 184.22±87.31^*^ |  | 8.84 | <0.001 |
| Stroop Tests | |  |  |  |  |  |  |  |
| Color congruent, ms | 959.3±227.6 |  | 1264.4±432.6^*^ |  | 1286.5±401.6^*^ |  | 15.76 | <0.001 |
| Color neutral, ms | 951.3±201.6 |  | 1243.9±395.1^*^ |  | 1262.4±389.4^*^ |  | 16.55 | <0.001 |
| Color incongruent, ms | 1221.0±325.4 |  | 1536.1±455.7^*^ |  | 1596.0±473.5^*^ |  | 14.30 | <0.001 |
| \| Lt=left; Rt=right; CDR=Clinical Dementia Rating Scale; MMSE= Mini Mental Status Examination; Est-RSPM=estimated Raven’s Standard Progressive Matrices; CVLT-2=California Verbal Learning Test, 2^nd^ ed.; BVMT-R =Brief Visual Memory Test-Revised; B3DCPT=Benton 3-D Constructional Praxis Test; PPT=Purdue Pegboard Test; VOSP-PD=Visual Object and Space Perception Battery-Position Discrimination; VOSP-NL= Visual Object and Space Perception Battery-Number Location.  *^a^*The global ratings for the CDR ranged from 0 to 0.5 point for all participants. \| \| --- \|   ^*^p<0.05 compared with the control group. | | | | | | | | |
